# To minimize foraging time, use high‐efficiency, energy‐expensive search and capture methods when food is abundant but low‐efficiency, low‐cost methods during food shortages

**DOI:** 10.1002/ece3.8204

**Published:** 2021-12-02

**Authors:** R. Åke Norberg

**Affiliations:** ^1^ Department of Biological and Environmental Sciences, Zoology University of Gothenburg Goteborg Sweden

**Keywords:** foraging modes, locomotion modes and energy expenditure, minimizing foraging time, optimal pursuit‐and‐capture methods, optimal search methods

## Abstract

Based on a mathematical model, I show that the amount of food in the habitat determines which among alternative methods for search of prey, respectively, for pursuit‐and‐capture give the shortest daily foraging time. The higher the locomotor activity, the higher the rate of energy expenditure and the larger the habitat space a predator can search for prey per time unit. Therefore, I assume that the more efficient a foraging method is, the higher its rate of energy expenditure. S*urvival selection* favors individuals that use foraging methods that cover their energy needs in the shortest possible time. Therefore, I take the optimization criterion to be minimization of the daily foraging time or, equivalently, maximization of the rate of net energy gain. When time is limiting and food is in short supply, as during food bottleneck periods, low‐efficiency, low‐cost foraging methods give shorter daily foraging times than high‐efficiency, energy‐expensive foraging methods. When time is limiting, food is abundant and energy needs are large, as during reproduction, high‐efficiency high‐cost foraging methods give shorter daily foraging times than low‐efficiency low‐cost foraging methods. When time is not limiting, food is abundant, and energy needs are small, the choice of foraging method is not critical. Small animals have lower rates of energy expenditure for locomotion than large animals. At a given food density and with similar diet, small animals are therefore more likely than large ones to minimize foraging time by using high‐efficiency energy‐expansive foraging methods and to exploit patches and sites that require energy‐demanding locomotion modes. *Survival selection* takes place at food shortages, while low‐efficiency low‐cost foraging methods are used, whereas *reproduction selection* occurs when food is abundant and high‐efficiency energy‐expensive foraging methods do better. In seasonal environments, selection therefore acts on different foraging methods at different times. Morphological adaptation to one method may oppose adaptation to another. Such conflicts select against foraging and morphological specialization and tend to give species‐poor communities of year‐round resident generalists. But a stable year‐round food supply favors specialization, niche narrowing, and dense species packing.

## 1 INTRODUCTION

Foraging consists of search followed by pursuit, capture and handling, or capture for short. Capture time is the time from the detection of a potential prey item to the point when it can be ingested or taken care of. Capture may include pursuit, bringing down, and killing of animal prey or extraction of other food resources. It is important to distinguish search from capture because an animal can search for all available prey simultaneously but captures them one at a time. The time, during which an animal pursuits and captures prey, is not available for search (Holling, 1965, 1968; MacArthur & Pianka, 1966; Schoener, 1971).

Search time increases with decreasing prey density, whereas capture time per item generally does not. The moment a predator detects a prey animal, the search phase ends, and the capture phase starts as the predator moves toward the prey over a distance that initially equals the detection distance. Time and energy expenditure is thereby added to the capture costs. The time required for capture is independent of prey density. This is the situation represented by the present model.

But food density may determine capture time if a forager's perceptual range is much larger the average distance between food items. Several food items may then be perceived simultaneously. The time required to move up to them is shorter the higher their density. Insects visiting flowers in a meadow are example of this.

Foraging animals can be categorized as “time minimizers” or “energy maximizers” (Schoener, 1974). Time minimizers minimize the foraging time required to cover their energy needs, whereas energy maximizers not only cover their basic needs but also maximize the amount of net energy acquired, using the surplus to gain weight, put up stores, or reproduce. An additional dichotomy distinguishes between “searchers” and “pursuers.” Even though there is a continuous range, “searchers” spend most of the foraging time searching for prey, whereas “pursuers” spend much time on pursuit, capture, and handling (MacArthur & Pianka, 1966).

Norberg (1977) drew attention to the large amount of energy that animals expend on locomotion for foraging to cover their basic needs and emphasized the accompanying increase in daily foraging time. Flight is particularly expensive in energy. Thus, level flight in the neotropical nectar‐feeding bat *Glossophaga soricina* has been estimated from quasi‐steady aerodynamic theory to expend metabolic energy at a rate 12 times the basal metabolic rate, BMR, whereas hovering flight may require 21 BMR (Norberg et al., 1993). The rate of metabolic energy expenditure has been measured to 6–8 BMR in level flight in the laughing gull *Larus atricillla* (Tucker, 1972), to 8.2 BMR in level flight in the rock dove *Columba livia* (LeFebvre, 1964), to 23 BMR in the European robin *Erithacus rubecula*, while making short (3 m) and brief (0.78 s) flights in an aviary at substantially lower speeds than the minimum power speed *V_mp_* (Tatner & Bryant, 1986), and to 11.7 BMR in the willow tit *Parus montanus* during short flights in an aviary at lower speeds than the *V_mp_* (Carlson & Moreno, 1992). Such short and slow flights are very common among birds that forage in dense vegetation and in tree canopies. Flight below cruising speed expends energy at higher rates than flight at the minimum‐power or maximum‐range speeds. Simpler and more sluggish locomotion modes may also raise energy expenditure considerably. The sea snail *Littorina littorea*, for example, increases its oxygen consumption 15 times the basal rate when crawling (Newell, 1970).

Animals usually have a repertoire of foraging methods, each of which involves a characteristic locomotion mode, food‐finding efficiency, and rate of energy expenditure. Here are some examples.

The European kestrel *Falco tinnunculus* spends most of its foraging time searching for food and can therefore be categorized a “searcher.” One of its two foraging modes is flight‐hunting, where it searches for prey during near‐hovering flight, termed “wind‐hovering,” by which it remains stationary relative to the ground by flying into prevailing winds at the wind speed. It requires less power than true hovering in still air but more than cruising flight (Videler et al., 1983; Videler, 2005). The other mode is perch‐hunting, where it searches from a perch and strikes from there.

A year‐round study in the Netherlands quantified the metabolic energy expenditure and rate of energy yield from alternative foraging methods of the kestrel (Masman et al., 1988). The rate of energy expenditure was estimated to be eight times higher for flight‐hunting than for perch‐hunting. But flight‐hunting gave 11 times more net energy gain per unit of time than perch‐hunting in winter and 30 times more than perch‐hunting in summer.

In summer, kestrels reduced foraging time by using the high‐yield high‐cost flight‐hunting technique during a larger proportion of the hunting time (32%) than in winter (19%). This is a move toward time minimization in summer when food is abundant.

Hummingbirds and honeyeaters may be labeled “pursuers” because most of their foraging time is spent flying between flowers and extracting nectar. Most hummingbirds, *Trochiliidae*, hover freely in front of flowers while feeding. Hovering is very expensive in energy but enables a bird to move more quickly between flowers than if it perches at the flowers it feeds from (Pyke, 1981). Australian honeyeaters, *Meliphagidae*, include nectar in their diet. They seldom hover but almost invariably perch while feeding. A honeyeater weighs on average three times more than a hummingbird, so hovering is much more expensive for honeyeaters, whereas their efficiency of nectar extraction is similar (Pyke, 1981). The large energy cost of hovering and the lack of compensation by a better nectar extraction capacity are likely reasons why honeyeaters use a low‐efficiency low‐cost foraging method rather than hovering. But the small body mass of hummingbirds keeps down their energy cost of hovering, which enables them to forage by hovering flight.

Based on a mathematical model, Norberg (1977) predicted that an animal can minimize foraging time by using high‐efficiency high‐cost search methods when food is abundant. But as food density decreases, it should switch to less energy‐expensive search methods despite their lower efficiency in finding food. Similar predictions have been made from quite different arguments (Evans, 1976) as well as from a theory based on detection probabilities (Andersson, 1981).

To test my earlier model (Norberg, 1977), I have recorded the frequency by which goldcrests *Regulus regulus* (L.) forage by hovering flight. Hovering flight furthers foraging efficiency but is extremely expensive in energy. As predicted, hovering frequently was high in autumn when arthropod prey was abundant in spruce trees *Picea abies* (L.), but very low in early spring when prey was scarce. Prey density index was 85 in autumn and 13 in spring. Goldcrests hovered 5.29 times per minute in autumn and 0.23 times per minute in spring, so prey density and hovering frequency were 6.5, respectively, 23 times higher in autumn than in spring (Norberg, 2021).

Norberg (1977) explored how food density in the habitat dictates the optimal choice between different *search methods* but did not consider whether food density also determines which among different *capture methods* is the best choice. During the goldcrest study, I found that it may be difficult to determine whether their hovering flight is attributable to search or to capture. Therefore, and because pursuers may spend more time pursuing, capturing and handling food than searching for it, I here present a more comprehensive model that treats both the search and capture phases of foraging and explores whether the amount of food in the habitat also determines which capture method gives the shortest daily foraging time.

## 2 ENERGY AND TIME EXPENDITURE FOR FORAGING

Time for foraging may be a resource in limited supply (Holling, 1968). It happens when prey density in the habitat decreases so that the foraging time required approaches the time available. Hunting on a declining prey population is a vicious cycle; the lower the prey density becomes, the more time and energy a predator must expend on foraging and the more food it needs. Individuals that are able to lower food density to levels at which others cannot sustain their energy needs may eliminate competitors by starvation. In such competitive situations, *survival selection* favors individuals that use time‐minimizing foraging methods, which enables them to cover their energy needs in the shortest possible time. Therefore, I take the optimization criterion to be minimization of the necessary daily foraging time or, equivalently, maximization of the rate of net energy gain.

Here, I consider a 24‐h energy budget and explore which among alternative foraging modes give the shortest daily foraging time when food is abundant as opposed to when it is scarce. Different foraging modes are described exclusively by two characteristics: *search methods* by their search efficiency and rate of energy expenditure and *capture methods* by the time taken for an average capture event and their rate of energy expenditure.

A fundamental premise is that the more efficient a *search method* is, the higher its rate of energy expenditure. A rationale for this is that the more actively an animal moves about, the higher its encounter rate with prey but the higher the rate of energy expenditure. A basic assumption when comparing different *capture methods* is that the shorter the time required to capture one average prey item, the higher the rate of energy expenditure. If a high‐efficiency search method, or a fast capture method, were also the least energy‐expensive, there would be no need for costlier alternatives. I take search time to vary in inverse proportion to food density in the habitat but regard time per capture event to be independent of it.

### 2.1 Daily energy expenditure

A minimal daily energy budget must cover the energy cost for basal metabolism and foraging activities. Rate of energy expenditure, or power, has dimension joule per second, which defines watt. Multiplication by time in seconds gives the amount of energy expended, expressed in joule. Foraging activities consist of a *search* phase, which turns into a *capture* phase when a prey item has been detected and the predator pursues and captures it. The number of food items *n_eat_* that an animal must eat in 24 h equals the total amount of energy expended divided by the amount of net energy *e* assimilable from an average food item,

(1) $$n_{\mathrm{eat}}=\frac{DP_{\mathrm{BMR}}+T_{s}p_{s}=n_{\mathrm{eat}}t_{c}p_{c}}{e}.$$

Here, *D* is the number of seconds in 24 h (24 × 3600 s), *P_BMR_* is the basal metabolic rate in watt, *DP_BMR_* is the metabolic energy cost in joules for basal metabolism in 24 h, *T_s_* is the daily time in seconds spent searching for food, *p_s_* is the rate of energy expenditure during search, *t_c_* is the average time in seconds required to capture one food item, and *p_c_* is the average rate of energy expenditure during a capture event, so *t_c_p_c_* is the average amount of energy expended for capturing one prey item. The power expenditures for search *p_s_* and for capture *p_c_* are in addition to *P_BMR_*. Rearrangement of terms in Equation 1 gives

(2) $$n_{\mathrm{eat}}=\frac{DP_{\mathrm{BMR}}+T_{s}p_{s}}{e‐t_{c}p_{c}}.$$

### 2.2 Daily search time

All potentially detectable food items are searched for simultaneously. The time *T_s_* required to search for and find the number *n_eat_* of prey items eaten in 24 h should therefore be inversely proportional to the number *N* of prey per unit of habitat area, and inversely proportional also to search efficiency, contained in *k_s_*, so from Equation 2:

(3) $$T_{s}=\frac{n_{\mathrm{eat}}}{Nk_{s}}=\frac{DP_{\mathrm{BMR}}+T_{s}p_{s}}{Nk_{s}\left(e‐t_{c}p_{c}\right)}.$$

The constant *k_s_* increases linearly with increasing efficiency of a search method in finding food. Search efficiency may depend on variables such as search behavior, locomotor mode and speed, visual resolution, auditory acuity, echolocation range, and so on. The higher the value of *k_s_*, the shorter the search time. The *k_s_* also expresses the proportionality between search time and the size of the unit habitat area used for expressing *N*. It is dimensionally consistent with Equation 3. Rearrangement of terms in Equation 3 gives:

(4) $$T_{s}=\frac{DP_{\mathrm{BMR}}/k_{s}}{N\left(e‐t_{c}p_{c}\right)‐p_{s}/k_{s}}.$$

**FIGURE 1:**
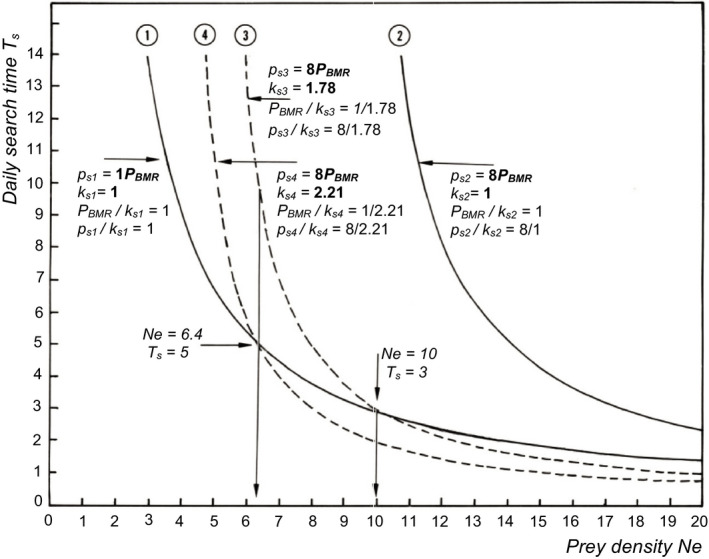
Curves generated from Equation 5 for each of four different *search* methods, labeled 1–4, showing daily search time *T_s_* as a function of prey density *Ne*. Time is expressed in hours, so *D* = 24 h. The *capture* method is the same in all cases, and the energy cost *t_c_p_c_* for capturing one average food item is set to 0.1*e*, so (1 – *t_c_p_c_*/*e*) =0.9 in Equation 5. **Method 1**: The rate *p_s1_* of energy expenditure for search is taken to equal 1*P_BMR_*—over and above *P_BMR_*. The ratio *P_BMR_*/*k_s1_* between the basal metabolic rate and the search efficiency is set to 1, so the ratio *p_s1_*/*k_s1_* between rate of energy expenditure and search efficiency also equals 1. **Method 2**: The rate *p_s_* of energy expenditure for search is 8*P_BMR_*, but the search efficiency *k_s2_* is taken to be identical to that of method 1, so *P_BMR_*/*k_s2_* = 1 and *p_s2_*/*k_s2_* = 8. Method 2 gives longer search times than the others at all food densities. **Method 3**: The rate *p_s_* of energy expenditure for search is 8*P_BMR_*, as with method 2, but the search efficiency *k_s3_* is 1.78 times better than with methods 1 and 2, so *P_BMR_*/*k_s3_* = 1/1.78 and *p_s3_*/*k_s3_* = 8/1.78. **Method 4**: The rate *p_s_* of energy expenditure for search is 8*P_BMR_*, like with methods 2 and 3, but the search efficiency *k_s4_* is 2.21 times better than with method 1 and 2, so *P_BMR_*/*k_s4_* = 1/2.21 and *p_s4_*/*k_s4_* = 8/2.21. When the rate of energy expenditure for search is increased eightfold, from 1*P_BMR_* in method 1 to 8*P_BMR_* in each of method 2, 3, and 4, the resulting prolongation of search time can be eliminated by modest increases in search efficiency *k_s_*. When food is abundant, curves for the high‐cost, high‐efficiency methods 3 and 4 pass below the curve for the low‐cost, low‐efficiency method 1. The intersections between curves 3 and 1 and between 4 and 1 show that for the high‐cost methods 3 and 4 to give the same daily search time as the low‐cost method 1, their search efficiency *k_s_* must be increasingly higher the lower the prey density (*k_s3_* =1.78 for intersection at *Ne*=10 and *k_s4_* =2.21 for intersection at *Ne*=6.4). When prey density is low, and since there is a limit to efficiency, low‐efficiency low‐cost methods are likely to give shorter daily search times than high‐cost high‐efficiency methods. The odd efficiency ratios 1.78 and 2.21 arose because I placed the intersection between curves 1 and 3 at *T* ***_s_*** =3 and that between curves 1 and 4 at *T* ***_s_*** =5

To avoid having to assign a value to *e* when making the graph in Figure 1, I express the energy cost *t_c_p_c_* of one average capture event in terms of a dimensionless ratio *t_c_p_c_*/*e*, which is the energy cost for capturing one average prey animal as a proportion of its energy content. Equation 4 can then be reformulated to show *T_s_* as a function of *Ne*, which is the total amount of energy contained in prey animals per unit habitat area,

(5) $$T_{s}=\frac{DP_{\mathrm{BMR}}/k_{s}}{Ne\left(1‐t_{c}p_{c}/e\right)‐p_{s}/k_{s}}.$$

### 2.3 Daily capture time

Prey items are captured and handled one at a time, so the average time taken to capture one average prey is independent of prey density. The *capture* phase may consist of pursuit, capture, and handling.

With the use of *T_s_* = *n_eat_*/*Nk_s_* in Equation 3, I eliminate *T_s_* from Equation 2 and rearrange terms:

(6) $$n_{\mathrm{eat}}=\frac{DP_{\mathrm{BMR}}N}{N\left(e‐t_{c}p_{c}\right)‐p_{s}/k_{s}}.$$

The daily time *T_c_* required to capture the necessary number *n_eat_* of prey items in 24 h equals *n_eat_* multiplied by the average time *t_c_* required to capture one prey,

(7) $$T_{c}=n_{\mathrm{eat}}t_{c}=\frac{DP_{\mathrm{BMR}}Nt_{c}}{N\left(e‐t_{c}p_{c}\right)‐p_{s}/k_{s}}.$$

## 3 OPTIMAL SEARCH METHOD AT DIFFERENT PREY DENSITIES

Different search methods vary in efficiency and rate of energy expenditure. They may involve lying in ambush, “sit‐and‐wait,” thermal soaring, running, cruising flight, and hovering flight, tactics listed here in order of increasing rate of energy expenditure. In general, the higher the locomotor activity, the higher the rate of energy expenditure and the larger the habitat space searched per unit of time. Therefore, I assume that the more energy a search method expends per unit of time the more efficient it is.

I have shown earlier that food availability determines which among alternative search methods result in the shortest daily search time (Norberg, 1977). Here, I present a modified analysis and compare two alternative search methods at a time. They are characterized exclusively by their respective search efficiency *k* ***_s_*** and rate of energy expenditure *p* ***_s_***. At a given prey density in the habitat, a low‐efficiency low‐cost method is regarded equally beneficial as a high‐efficiency high‐cost method if both result in the same daily search time.

Note that both the daily search time *T* ***_s_*** in Equation 5 and the daily capture time *T* ***_c_*** in Equation 7 are functions of the search efficiency *k* ***_s_***, rate *p* ***_s_*** of energy expenditure during search, time *t* ***_c_*** needed to capture one average food item, and rate *p_c_* of energy expenditure during a capture event. Because the energy expended on search influences the number of prey items that must be eaten, the choice of search method determines not only the daily search time but also the daily capture time. Likewise, the choice of capture method affects energy expenditure and daily capture time as well as daily search time.

Figure 1 is based on Equation 5 and shows daily search time *T_s_* versus *Ne* for each of four different search methods, where *Ne* is prey energy content per unit habitat area. I divide both sides of Equation 5 by 3600 to get *D* equal to 24 h and *T_s_* expressed in hours. The numerical values of the variables in Equation 5 are chosen such that the resulting daily search times are realistic and range from 0 to 14 h in a 24‐h day.

The characteristics of search method 1 are specified in the legend to Figure 1. Each one of search methods 2, 3, and 4 is taken to consume eight times more energy per unit of time than method 1. The search efficiency is set to 1 for methods 1 and 2. But it is 1.78 times better for method 3 and 2.21 times better for method 4.

Which one of methods 1, 3, and 4 that gives the shortest search time depends on prey density in the habitat. For illustrative purposes, I let curves 1 and 3 intersect at *T_s_* =3, whereas curves 1 and 4 intersect at *T_s_* = 5. This is the reason for the odd values 1.78 and 2.21 of the efficiency ratios *k_s3_*/*k_s1_* and *k_s4_*/*k_s1_*. They were found from Equation 9 by letting *T_s_* =3, *p_s1_*/*P_BMR_* =1, and *p_s3_*/*P_BMR_* =8 and solving for *k_s3_*/*k_s1_*, yielding 1.78, and by letting *T_s_* =5, *p_s1_*/*P_BMR_* =1, and *p_s4_*/*P_BMR_* =8, and solving for *k_s4_*/*k_s1_*, yielding 2.21. The *Ne* coordinates corresponding to the *T_s_* intersection points at 3 and 5, respectively, were then found by inserting the relevant values of *T_s_*, *P_BMR_*/*k_s_*, and *p_s_*/*k_s_* in Equation 5, and solving for *Ne*.

In Figure 1, method 3 expends eight times more energy per unit time than method 1 but because it is 1.78 times more efficient, it gives 29% shorter daily search time *T_s_* when *Ne* = 20 and the same search time as method 1 when *Ne* =10. Method 4 also expends eight times more power than method 1, but it is 2.21 times more efficient and therefore gives 46% shorter daily search time than method 1 when *Ne* = 20 and 33% shorter search time than methods 1 and 3 when *Ne* = 10. When *Ne* = 6.4, methods 1 and 4 perform equally and both give 52% shorter search time than method 3. The low‐cost low‐efficiency method 1 outperforms the high‐cost high‐efficiency method 3 when *Ne* is less than 10, and it outperforms method 4 when *Ne* is less than 6.4.

An energetic disadvantage, due to a high rate of energy expenditure, can thus be offset by a modest increase in search efficiency. But the lower the food availability—and the longer the daily search time—the better the search efficiency must be to compensate for a given energetic disadvantage. Since there is a limit to how efficient a method can be, and given that time minimization is the goal, high‐efficiency high‐cost search methods do better than low‐efficiency low‐cost methods when food is abundant, whereas low‐efficiency low‐cost methods are superior when food is in short supply.

**FIGURE 2:**
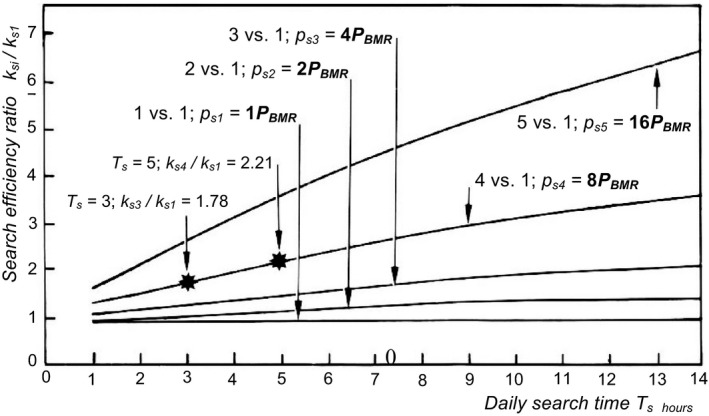
Curves generated from Equation 9 showing how the ratio *k* ***_si_***/*k* ***_s1_*** between the *search* efficiency *k* ***_si_*** of method *i* and *k* ***_s1_*** of method 1 must vary as a function of the daily search time *T* ***_s_*** to compensate for the higher rate of energy expenditure of method *i* such that both result in the same daily search time. To be able to keep within a realistic range of food density, I have converted food density to corresponding search times. The daily search time *T* ***_s_*** increases from left to right because of decreasing food abundance *Ne*. Time *T_s_* is expressed in hours, so *D* = 24 h in Equation 9. Each curve represents a comparison between two *search* methods. Method 1 is a reference, against which methods 2–5 are compared, one at a time. The *capture* method is the same in all comparisons. *Search* method 1 is taken to use energy at a rate *P_s1_* equal to 1*P_BMR_*—over and above *P_BMR_*—in all comparisons. Search methods 2–5 are more expensive and use up energy at the respective rates 2*P_BMR_*, 4*P_BMR_*, 8*P_BMR_*_,_ and 16*P_BMR_*. For any *k* ***_si_***/*k* ***_s1_*** ratio read off a curve, *k* ***_si_*** is just large enough to compensate for the higher energy expenditure of search method *i*, such that it gives the same daily search time *T* ***_s_*** as the low‐cost method 1. For any combination of *k* ***_si_***/*k* ***_s1_*** and *T* ***_s_*** above a curve, the efficiency *k* ***_si_*** of the high‐cost method *i* is better than required to compensate for its higher energy expenditure, so it gives shorter daily search times than method 1. But for coordinates below a curve, *k* ***_si_*** is not large enough, so the low‐efficiency, low‐cost method 1 gives shorter daily search times. High‐efficiency, high‐cost methods thus tend to be superior when search times are short owing to high food density in the habitat. But since there is a limit to how high the efficiency *k* ***_si_*** can be, low‐efficiency, low‐cost methods are likely to be superior when search times are long due to food shortage. The highlighted *T* ***_s_***/(*k* ***_si_***/*k* ***_s1_***) coordinates 3/1.78 and 5/2.21 refer to Figure 1 and correspond to the intersection between curves 1 and 3 at *Ne* =10 and *T* ***_s_*** =3, and between curves 1 and 4 at *Ne* =6.4 and *T* ***_s_*** =5. The odd values of the *k_si_*/*k_s1_* ratios arose because I placed the intersection point between curves 1 and 3 in Figure 1 at *T* ***_s_*** =3, and that between curves 1 and 4 at *T* ***_s_*** =5

Next, I will compare two search methods, labeled 1 and 2. Method 2 is taken to be more expensive in energy than method 1. I will examine how much the efficiency *k_s2_* of method 2 must exceed the efficiency *k_s1_* of method 1 to compensate for its higher energy expenditure, such that both result in the same daily search time at a given food density in the habitat. But since any size can be chosen for the unit habitat area used for expressing food density, one cannot identify any meaningful *Ne* range against which to plot the critical efficiency ratio *k_si_*/*k_s1_* that compensates for the higher rate of energy expenditure of method 2. However, the time actually available for foraging, such as number of daylight hours, defines a meaningful range of *T_s_* and is therefore chosen as the independent variable here and in Figure 2.

I want to express the ratio *k_s2_*/*k_s1_* as a function of *p_s1_*, *p_s2_*_,_ and *T* ***_s_***. In the following comparisons, each of the alternative *search* methods is combined with the same *capture* method, so *t* ***_c_*** and *p_c_* are kept constant. To obtain *k_s2_*/*k_s1_* as a function of *T_s_* rather than of *N*, I solve Equation 4 with respect to *N* for each of search methods 1 and 2, identified by subscripts:

(8a) $$N_{s1}=\frac{DP_{\mathrm{BMR}}/k_{s1}+T_{s1}p_{s1}/k_{s1}}{T_{s1}\left(e‐t_{c}p_{c}\right)},$$

(8b) $$N_{s2}=\frac{DP_{\mathrm{BMR}}/k_{s2}+T_{s2}p_{s2}/k_{s2}}{T_{s2}\left(e‐t_{c}p_{c}\right)}.$$

Two search methods are regarded equally beneficial at a given food density when they result in the same daily search time. Therefore, I let *T_s1_* = *T_s2_* and *N_s1_* = *N_s2_*, equate Equations 8a and 8b, and solve for *k_s2_*/*k_s1_*, whereby *e* and the terms *t_c_* and *p_c_* for capture cancel out:

(9) $$\frac{k_{s2}}{k_{s1}}=\frac{D+T_{s}p_{s2}/P_{\mathrm{BMR}}}{D+T_{s}p_{s1}/P_{\mathrm{BMR}}}.$$

Power has dimension energy/time (joule/s). But to convert daylength *D* and search time *T_s_* to hours, I divide the numerator and denominator in Equation 9 by 3600, whereas the ratios *p_s2_*/*P_BMR_* and *p_s1_* /*P_BMR_* remain dimensionless.

Figure 2 shows the behavior of Equation 9. Each curve represents a comparison between two search methods with different rates of energy expenditure *p_s_*, expressed as multiples of *P_BMR_*. Method 1 is assumed to expend energy at rate *p_s1_* = 1*P_BMR_*, so *p_s1_* /*P_BMR_* =1. It is used as a reference, against which four more expensive methods are compared, one at a time, expending energy at the respective rates 2*P_BMR_*, 4*P_BMR_*, 8*P_BMR_*, and 16*P_BMR_*. Energy expenditure *p_s1_* of method 1 is kept equal to 1*P_BMR_* in all pairwise comparisons. Each of the alternative high‐cost search methods gives equally long search time *T_s_* as does method 1 when the *k_si_*/*k_s1_* ratio varies with *T_s_* as shown by the respective curve. Equation 9 and Figure 2 thus define what the *k_si_*/*k_s1_* ratio between two alternative search methods must be to compensate for the higher energy expenditure of method *i*, such that curves in a diagram showing *T_s_* versus *Ne* for each of the two search methods intersect at a given value of *T_s_* and the corresponding *Ne* coordinate (Figure 1).

As one moves rightward along a curve in Figure 2, the daily search time becomes longer because of decreasing prey density, and the *k_si_*/*k_s1_* ratio must be progressively larger to compensate for the higher energy consumption rate *p_si_* of method *i*, such that it results in the same daily search time *T_s_* as method 1 at a given prey density *Ne*. For coordinates above a given curve, the *k_si_*/*k_s1_* ratio is larger than required to compensate for the higher rate of energy expenditure of method *i*, so method *i* gives shorter daily search times than the low‐efficiency low‐cost method 1. But for coordinates below a curve, the *k_si_*/*k_s1_* ratio is not large enough to outbalance the higher energy cost *p_si_* of method *i*, so method 1 gives shorter daily search times than method *i*.

Since there is a limit to how efficient a search method can be, a general conclusion is that when prey is abundant, and the daily search time therefore is short (to the left in Figure 2), high‐efficiency high‐cost methods are likely to give shorter daily search times than low‐efficiency low‐cost methods. But when prey density is low, which necessitates long daily search times (to the right in Figure 2), low‐efficiency low‐cost methods are superior and tend to give shorter daily search times than high‐efficiency high‐cost methods.

## 4 INCREASED ENERGY DEMANDS

Extra energy expenditure, such as for thermoregulation or reproduction, increases the metabolic rate and can be accounted for by multiplying *P_BMR_* in Equation 5 by an appropriate factor. The curves in Figure 1, showing *T_s_* versus *Ne*, and the intersection point between curves 1 and 3 and that between curves 1 and 4 will then be displaced vertically upward by that factor, so the cross‐over from one method to another occurs at the same prey density *Ne* in the habitat regardless of the amount of extra food and search time *T_s_* required.

As an example, we consider the intersection point between curves 1 and 3 in Figure 1. It occurs at *Ne* =10 and *T_s_* =3 h. The cross‐over at *Ne* =10 requires that the efficiency ratio *k_s3_*/*k_s1_* equals 1.78, meaning that method 3 is 1.78 times more efficient than method 1 (Figures 1 and 2).

Then, we assume that the rate of energy expenditure, averaged over a 24‐h period, is increased by 2*P_BMR_*. It corresponds to multiplying Equation 5 by 3. The intersection between the curves for method 1 and 3 still occurs at *Ne* =10 but the search time triples, from 3 to 9 h. The efficiency ratio *k_s3_*/*k_s1_* is unchanged (Figures 1 and 2).

The increase in daily capture time *T_c_*, caused by extra energy expenditure, can be found likewise by multiplying *P_BMR_* in Equation 7 by an appropriate factor.

## 5 OPTIMAL CAPTURE METHOD AT DIFFERENT PREY DENSITIES

Pursuit‐and‐capture methods vary in efficiency and consist of different locomotor modes, such as a short attack after waiting in ambush, long‐distance running, flying pursuit, or hover‐capture, locomotor modes listed here in order of increasing rate of energy expenditure. The efficiency of a capture method is inversely proportional to the time required to catch one average food item after detection. Similar to assumptions about search methods, I assume that a quick capture method expends energy at a higher rate than slower methods. If instead a low‐cost capture method were also the fastest, there would be no need for costlier alternatives. To explore whether the optimal choice of capture method varies with food density in the habitat, I will compare two capture methods at a time, one of which is faster than the other but expends energy at a higher rate than the slow method.

I compare capture methods 1 and 2, each characterized exclusively by the average time *t_c_* required to capture one prey item and the average rate *p_c_* of energy expenditure during a capture event. Method 2 is taken to require more power than method 1. I will examine how much shorter the capture time *t_c2_* of method 2 must be to compensate for its higher rate *p_c2_* of energy expenditure, such that both result in the same daily capture time. Similar to the analysis of search times, I want to express the ratio *t* ***_c_*** *_2_*/*t* ***_c_*** *_1_* as a function of *p_c1_*, *p_c2_*, and *T_c_*. In all comparisons between *capture* methods the *search* method is the same, so *p_s_* and *k_s_* remain constant. To obtain *t* ***_c_*** *_2_*/*t* ***_c_*** *_1_* as a function of *T_c_* rather than of *N*, I solve Equation 7 with respect to *N* for each capture method, identified by subscripts:

(10a) $$N_{c1}=\frac{T_{c1}p_{s}/k_{s}}{T_{c1}e‐T_{c1}t_{c1}p_{c1}‐t_{c1}DP_{\mathrm{BMR}}},$$

(10b) $$N_{c2}=\frac{T_{c2}p_{s}/k_{s}}{T_{c2}e‐T_{c2}t_{c2}p_{c2}‐t_{c2}DP_{\mathrm{BMR}}}.$$

The two capture methods are equally beneficial at a given number *N* of prey per unit habitat area when they result in the same daily capture time *T_c_*. Therefore, I let *T_c1_* = *T_c2_* and *N_c1_* = *N_c2_* and equate Equations 10a and 10b and solve for *t_c2_* /*t_c1_*, whereby *e* and the terms *k_s_* and *p_s_* for search cancel out:

(11) $$\frac{t_{c2}}{t_{c1}}=\frac{D+T_{c}p_{c1}/P_{\mathrm{BMR}}}{D+T_{c}p_{c2}/P_{\mathrm{BMR}}}.$$

Power has dimension energy/time (joule/s). But to convert daylength *D* and capture time *T_c_* to hours, I divide the numerator and denominator in Equation 9 by 3600, whereas the ratios *p_c2_*/*P_BMR_* and *p_c1_*/*P_BMR_* remain dimensionless. The time *t_c_* required to capture one average prey item is independent of prey density. But when food in the habitat decreases, the daily *search* time *T_s_* increases, so more energy is expended on search. More prey items must therefore be eaten, which requires longer daily capture time *T_c_*.

**FIGURE 3:**
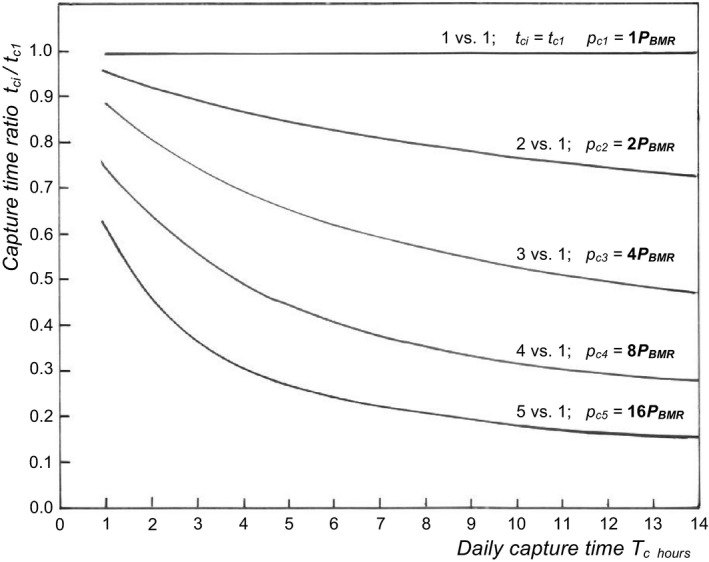
Curves generated from Equation 11, showing how the ratio *t* ***_ci_*/** *t* ***_c1_*** between the time *t* ***_ci_*** taken for an average *capture event* with method *i* and the time *t* ***_c1_*** taken with method 1 must vary as a function of the daily *capture* time *T_c_* to compensate for the higher rate of energy expenditure of method *i* such that it results in the same daily capture time as method 1. To be able to keep within a realistic range of food density, I have converted food density to corresponding capture times. The daily capture time *T* ***_c_*** increases from left to right because of decreasing food abundance *Ne*. Time *T_c_* is expressed in hours, so *D* = 24 h in Equation 11. Each curve represents a comparison between two *capture* methods. Method 1 is used as a reference, against which methods 2–5 are compared, one at a time. The *search* method is the same in all comparisons. Capture method 1 is assumed to use energy at a rate *P_c1_* equal to 1*P_BMR_*—over and above *P_BMR_*—in all comparisons. Capture methods 2–5 are more expensive and use up energy at the respective rates 2*P_BMR_*, 4*P_BMR_*, 8*P_BMR_*, and 16*P_BMR_*. For any *t* ***_ci_*/** *t* ***_c1_*** ratio read off a curve, *t* ***_ci_*** is just short enough to compensate for the higher energy expenditure of capture method *i*, such that it gives the same daily capture time *T* ***_c_*** as the slow low‐cost method 1. For any combination of *t* ***_ci_*/** *t* ***_c1_*** and *T* ***_c_*** below a curve, the expensive capture method *i* is faster than required to compensate for its higher energy expenditure, so it gives shorter daily capture times than method 1. But for coordinates above a curve, *t* ***_ci_*** is not short enough so the slow and low‐cost method 1 gives shorter daily capture times. When food is abundant—and daily capture times short—fast, high‐cost methods tend to give shorter daily capture times than slow low‐cost methods. But when food is in short supply, and since there is a limit to how short a capture event can be, slow low‐cost methods tend to give shorter daily capture times than fast methods

Figure 3 is based on Equation 11. Each curve represents a comparison between two capture methods with different rates of energy expenditure, expressed as multiples of *P_BMR_*. Method 1 is assumed to consume energy at rate *p_s1_* = 1*P_BMR_*. It is used as a reference against which four more expensive methods are compared, one at a time, expending energy at the respective rates 2*P_BMR_*, 4*P_BMR_*, 8*P_BMR_*, and 16*P_BMR_*. When *p_c1_* of method 1 is kept equal to 1*P_BMR_* in all comparisons, each of capture methods 1–4 results in the same daily capture time as method 1 when the ratio *t_ci_*/*t_c1_* varies with the daily capture time *T_c_* as shown by the curves for different rates *p_ci_* of energy expenditure during a capture event.

As one moves rightward along a curve in Figure 3, the daily capture time becomes longer. This is a consequence of decreasing prey density in the habitat. It necessitates longer search times and more energy expenditure, so more prey items must be captured, which takes more time. As capture time increases due to decreasing prey density, the *t_ci_*/*t_c1_* ratio must be ever smaller to compensate for the higher rate *p_ci_* of energy expenditure of method *i*, such that it results in the same daily capture time as method 1. For coordinates below a given curve, method *i* is faster than required to compensate for its higher rate of energy expenditure, so it gives shorter daily capture times than the low‐efficiency low‐cost method 1. But for coordinates above a curve, the *t_ci_*/*t_c1_* ratio is not small enough to outweigh the higher rate of energy expenditure *p_ci_* of method *i*, so method 1 is the better choice.

Since there is a limit to how short a capture event can be, a general conclusion is that when prey is abundant, and the daily capture time therefore is short (to the left in Figure 3), fast and high‐cost capture methods give shorter daily capture times than slow low‐cost methods. But when prey density is low, which necessitates long daily capture times (to the right in Figure 3), slow and low‐cost methods are superior and tend to give shorter daily capture times than fast high‐cost methods.

Equation 11 for capture methods and Equation 9 for search methods are similar except that the coefficient *k_s_* is directly proportional to search efficiency, whereas capture efficiency is higher the shorter the time *t_c_* required to capture one average food item after detection. Therefore, the ratio *p_c2_* /*P_BMR_* for the energy‐expensive *capture* method is in the denominator of Equation 11, while the corresponding ratio *p_s2_*/*P_BMR_* for the energy‐expensive *search* method is in the numerator of Equation 9. As a result, the capture time ratio *t_ci_*/*t_c1_*, which gives equal daily capture time, is a decreasing function of the daily capture time *T_c_* in Figure 3, whereas the efficiency ratio *k_s2_*/*k_s1_*, which gives equal search time, is an increasing function of *T_s_* in Figure 2.

## 6 CONCLUSIONS

1. Alternative search and capture foraging methods are characterized here exclusively by their respective combination of efficiency and rate of energy expenditure. I assume that the higher the efficiency of a foraging method, the higher its rate of energy expenditure. The optimization criterion is taken to be minimization of the daily foraging time, or equivalently, maximization of net energy gain per unit time. Which one among alternative foraging methods that is the best choice depends on prey density in the habitat. Some of the conclusions below were presented by Norberg (1977) but concerned only the search phase of foraging. The more comprehensive model presented herein generates the following conclusions, which apply equally to the search and to the capture phases of foraging.
2. *Time*‐*minimizing foraging methods should be used when the time required for foraging approaches the time available*. Hunting on a decreasing prey population is a vicious cycle; the lower the prey density becomes, the more time and energy a predator must expend on foraging and the more food it needs.
3. *When time is limiting and food is in short supply*, *as during food bottleneck periods*, *low*‐*efficiency low*‐*cost foraging methods give shorter daily foraging times than high*‐*efficiency energy*‐*expensive foraging methods*. Elimination by exploitation competition occurs when an individual is able to satisfy its energy needs, while food density is reduced to levels that others cannot survive. S*urvival selection* favors individuals that minimize foraging time by using low‐efficiency low‐cost foraging methods at food shortages.
4. *When time is limiting*, *food is abundant and energy needs are large*, *as during reproduction*, *high*‐*efficiency energy*‐*expensive foraging methods give shorter daily foraging times than low*‐*efficiency low*‐*cost foraging methods*. Figure 1 shows that considerable time can be saved by shifting foraging method when food density changes. *Reproduction selection* favors individuals that minimize the daily foraging time by using high‐efficiency energy‐expensive foraging methods when food is abundant, which maximizes daily net energy gain and reproductive output.
5. *When time is not limiting*, *food is abundant*, *and energy needs are small*, *the choice of foraging method is not critical*. When a small fraction of the available time is needed for foraging, there may be little incentive to minimize daily foraging time by using high‐efficiency energy‐expensive methods. Minimization of daily energy expenditure is then an alternative. But high‐efficiency energy‐expensive foraging methods would save time for other activities.
6. *At a given food density and with similar diet*, *small animals are more likely than large ones to use high*‐*efficiency energy*‐*expensive foraging methods and to exploit patches and sites that require energy*‐*demanding locomotion modes*. The energy expenditure for a given locomotor mode increases with increasing body mass. But it is not compensated for by increased foraging efficiency because agility and maneuverability decrease with increasing body size (Andersson & Norberg, 1981).
7. *Specialization and species packing*. When food fluctuates seasonally, the shortest daily foraging time will likely be achieved with different foraging methods in different seasons. During food shortages, *survival selection* acts to improve adaptations of low‐efficiency low‐cost foraging methods but when food is abundant *reproduction selection* favors adaptations of high‐efficiency energy‐expensive methods. Morphological adaptation to one method may oppose adaptation to another. Such conflicts select against foraging and morphological specialization and tend to give species‐poor communities of generalist year‐round residents. But a stable year‐round food supply favors specialization, niche narrowing, and dense species packing.

## 7 LIST OF SYMBOLS

**Table:** 

*e*	net energy assimilable from one average food item.
*k_s_*	a constant that expresses the efficiency of a search method in finding food and also sets the proportionality between search time and size of the unit habitat area chosen for expressing food density.
*N*	number of food items per unit habitat area.
*N_ci_*	number of food items per unit habitat area with capture method *i*.
*N_si_*	number of food items per unit habitat area with search method *i*.
*Ne*	food energy present per unit habitat area.
*n_eat_*	number of food items that an animal must eat to cover its entire 24‐h energy budget.
*P_BMR_*	basal metabolic rate (metabolic power; joule/second = watt).
*p_c_*	average rate of energy expenditure during a capture event (power; joule/second = watt), over and above the basal metabolic rate *P_BMR_*.
*p_s_*	average rate of energy expenditure during search for food (power; joule/second = watt), over and above the basal metabolic rate *P_BMR_*.
*D*	day length expressed in seconds (24 × 3600). Multiplication by the basal metabolic rate P_BMR_ gives the energy cost of basal metabolism in 24 h, exclusive of energy costs for foraging. When 3600 cancels in an equation, day length D = 24 h.
*T_c_*	total time in seconds that an animal must spend on pursuit, capture and handling of prey to cover its entire 24‐h energy budget. When 3600 cancels in an equation, *T_c_* is expressed in hour.
*T_s_*	total time in seconds that an animal must spend searching for prey to cover its entire 24‐h energy budget. When 3600 cancels in an equation, *T_s_* is expressed in hour.
*t_c_*	time in seconds that it takes to pursue, capture, and handle one average food item.

## CONFLICT OF INTERESTS

There are no conflicting interests.

## AUTHOR CONTRIBUTION

**Åke Rolf Norberg:** Conceptualization (equal); Data curation (equal); Formal analysis (equal); Funding acquisition (equal); Investigation (equal); Methodology (equal); Project administration (equal); Resources (equal); Software (equal); Supervision (equal); Validation (equal); Visualization (equal); Writing‐original draft (equal); Writing‐review & editing (equal).
